# Investigations of the novel checkpoint kinase 1 inhibitor SRA737 in non-small cell lung cancer and colorectal cancer cells of differing *tumour protein 53* gene status

**DOI:** 10.37349/etat.2023.00193

**Published:** 2023-12-21

**Authors:** Ali JN Duabil, Christian R Cooper, Esraa Aldujaily, Sarah ER Halford, Sandra Hirschberg, Sidath D Katugampola, George DD Jones

**Affiliations:** Istituto Nazionale Tumori-IRCCS-Fondazione G. Pascale, Italy; ^1^Leicester Cancer Research Centre, Department of Genetics & Genome Biology, University of Leicester, LE1 7RH Leics, UK; ^2^Department of Surgery, Faculty of Medicine, University of Kufa, Najaf, Iraq; ^3^MRC Oxford Institute for Radiation Oncology, University of Oxford, OX3 7DQ Oxon, UK; ^4^Department of Pathology & Forensic Medicine, Faculty of Medicine, University of Kufa, Najaf, Iraq; ^5^Cancer Research UK Centre for Drug Development, London E20 1JQ, UK

**Keywords:** Deoxyribonucleic acid damage, checkpoint kinase 1 inhibition, SRA737, comet assay, phosphorylated H2A.X variant histone

## Abstract

**Aim:**

In response to DNA damage the serine/threonine-specific protein kinase checkpoint kinase 1 (CHK1) is activated allowing cells to enter S phase (S) and G2 phase (G2) cell-cycle arrest. CHK1 inhibitors are expected to prevent cells from entering such arrest, thereby enhancing DNA damage-induced cytotoxicity. In contrast, normal cells with intact ataxia–telangiectasia mutated (ATM), CHK2 and tumour suppressor protein 53 (P53) signalling are still able to enter cell-cycle arrest using the functioning G1/S checkpoint, thereby being rescued from enhanced cytotoxicity. The main objective of this work is to investigate the *in vitro* effects of the novel CHK1 inhibitor SRA737 on pairs of non-small cell lung cancer (NSCLC) and colorectal cancer (CRC) cell lines, all with genetic aberrations rendering them susceptible to replication stress but of differing tumour protein 53 (*TP53*) gene status, focusing on DNA damage induction and the subsequent effects on cell proliferation and viability.

**Methods:**

NSCLC cell lines H23 [*TP53* mutant (MUT)] and A549 [*TP53* wild-type (WT)] and CRC cell lines HT29 (*TP53* MUT) and HCT116 (*TP53* WT) were incubated with differing micromolar concentrations of SRA737 for 24 h and then analysed using alkaline comet and phosphorylated H2A.X variant histone (γH2AX)-foci assays to assess mostly DNA single strand break and double strand break damage, respectively. Cell-counting/trypan blue staining was also performed to assess cell proliferation/viability.

**Results:**

Clear concentration-dependent increases in comet formation and γH2AX-foci/cell were noted for the *TP53* MUT cells with no or lower increases being noted in the corresponding *TP53* WT cells. Also, greater anti-proliferative and cell killing effects were noted in the *TP53* MUT cells than in the *TP53* WT cells.

**Conclusions:**

This study’s data suggests that P53 status/functioning is a key factor in determining the sensitivity of NSCLC and CRC cancer cells towards CHK1 inhibition, even in circumstances conducive to high replicative stress.

## Introduction

Maintaining low levels of DNA damage is essential for a cell’s normal/regulated proliferation and long-term survival [1]. DNA damage can occur from either endogenous processes, such as metabolic oxidative stress or replication errors, or from exogenous genotoxic sources, such as the anti-cancer treatments of radiotherapy and genotoxic chemotherapy. As part of their DNA damage response (DDR), cells respond to genomic damage by activating several cell-cycle checkpoints [including the G1 phase (G1)/S phase (S), S, and G2/M phase (M) checkpoints] enabling cycle arrest plus DNA repair, or programmed cell death [2, 3]. Consequently, this damage response can potentially limit the treatment efficacy of both radiotherapy and chemotherapy [4–6].

DDR to lethal immediate/prompt double-strand break damage is mostly facilitated through the phosphoinositide 3-kinase related kinase family member ataxia–telangiectasia mutated (ATM), whilst DDR to single-strand damage [including single strand breaks (SSBs) & stalled replication forks] is mostly mediated through the ataxia-telangiectasia and Rad3-related (ATR) protein [7]. Activated ATM leads to downstream phosphorylation/activation of checkpoint kinase 2 (CHK2) signalling, whilst ATR activation leads to activation of CHK1. The key function of these checkpoint kinases is to prevent DNA damage-bearing cells from continued further growth until repair is achieved [7].

In response to DNA damage, CHK1 has been shown to block the expression and function of cell division cycle 25 A (CDC25A) and CDC25C, allowing cells to enter S and G2 cell-cycle arrest respectively [4, 8, 9]. CHK1 also plays a key role in the S checkpoint preventing replication fork collapse [4, 10, 11] and also in homologous recombination repair via RAD51 homolog (RAD51) activation [12].

Many tumour cells have been shown to have inadequate DDR, with many harbouring flaws in key genes governing the cell-cycle checkpoints and repair. In turn, this can result in elevated levels of DNA damage and so contribute to tumorigenesis/progression [13]. Tumour suppressor protein 53 (P53) is frequently decontrolled in human tumour cells leading to compromised G1/S arrest and DNA repair [14–16]. Whilst these flaws can provide these cells with selective growth advantages (enabling adaptation, resistance and survival), these same cells will become ever more reliant on the remaining DDR factors and functional checkpoints to maintain viability. Therefore, an attractive therapeutic strategy to target cancer cells deficient in certain aspects of DDR is to intentionally target the remaining DDR pathways. By targeting these remaining DDR components one can achieve “synthetic lethality” inducing irreparable levels of lethal damage specifically in cancer cells of a predisposing genetic background. This tactic has been validated in cancer patients with mutations in breast cancer gene 1 (*BRCA1*) and *BRCA2* by the success of single agent poly (ADP-ribose) polymerase (PARP) inhibitor therapy [17].

Given the central role of CHK1 in DDR network signalling, the targeted inhibition of CHK1 provides various opportunities to achieve synthetic lethality in specific tumour genotypes; notably, in tumour cells exhibiting loss-of-function mutations in tumour protein 53 (*TP53*) or *ATM* or in other repair genes. In this circumstance, CHK1 inhibitors are expected to prevent cells from entering S and G2/M arrest, thereby enhancing DNA damage and cytotoxicity. In contrast, normal cells or cancer cells with intact ATM, CHK2 and P53 signalling will still be able to enter cell-cycle arrest using the functioning G1/S checkpoint, thereby being rescued from the enhanced cytotoxicity [18–21]. Furthermore, studies suggest that myelocytomatosis (MYC) and rat sarcoma (RAS) activation leads to heightened replication stress caused by increased firing of origins of replication [1, 2, 22, 23]. Accordingly, given the key role of CHK1 in maintaining fork stability, CHK1 inhibition may be most efficacious in tumours with higher replication stress levels. Two common tumour indications of high unmet medical need, notably in the advanced setting, with a high prevalence of the above-mentioned genetic aberrations include metastatic colorectal and non-small cell lung cancer (NSCLC).

Colorectal cancer (CRC) is the 3rd most common cancer worldwide, with an estimated 1.93 million new cases diagnosed in 2020 causing 0.94 million deaths worldwide [24]. In the UK approximately a quarter of sufferers have metastatic disease at the time of diagnosis and this contributes to CRC being regarded as the second most lethal cancer [25]. Furthermore, for those patients treated curatively, approximately 50% will suffer a relapse within 24 months [26]. Reported median overall survival in patients participating in clinical trials has increased to 30 months, however real world data indicates median overall survival may be closer to 12 months [27], this is despite advances in systemic therapies such as monoclonal antibodies to vascular endothelial growth factor (VEGF; bevacizumab), epidermal growth factor receptor (EGFR; cetuximab and panitumumab), v-raf murine sarcoma viral oncogene homolog B1 (BRAF; encorafenib), and immunotherapies (e.g. ipilimumab, nivolumab, and pembrolizumab) which are available to subsets of patients. However, genetic mutations in CRC such as Kirsten RAS (*KRAS*), *TP53* or mismatch repair (MMR) genes allow for replication errors and instability, making CRC an ideal cancer for CHK1 inhibition.

Lung cancer is the second commonest cancer worldwide, with more than 2.2 million cases and nearly 1.8 million deaths being estimated in 2020 [28]. The predominant form of lung cancer is NSCLC which accounts for 70–80% of all lung cancers. Unfortunately, the majority of sufferers are diagnosed with advanced disease, and despite recent advances in treatment, such as targeted [29–31] and immunotherapy-combination therapies [32], many patients do not achieve long-term control. Consequently, the development of new secondary treatments is a key area for future medical research. However, similar to CRC, genetic mutations in tumour suppressor genes such as *TP53* and cyclin-dependent kinase inhibitor 2a (*CDKN2A*), activating mutations in oncogenic drivers such as *MYC*, *KRAS*, cyclin E1 (*CCNE1*) and mutations in *ATR* and *BRCA1 & 2*, all make NSCLC a potentially ideal cancer for CHK1 inhibition.

SRA737 (formerly known as CCT245737) is an effective, highly selective, orally administered small molecule CHK1 inhibitor, that was produced via a collaboration between Cancer Research Technology Ltd., the Institute of Cancer Research Cancer Therapeutics Unit and Sareum Ltd. and then by Cancer Research UK with funding from the Cancer Research Technology Pioneer Fund managed by Sixth Element Capital. Subsequently, Sierra Oncology Inc. then acquired SRA737 in September 2016. Following the acquisition of Sierra Oncology by GlaxoSmithKline in July 2022, the rights to SRA737 were returned to Sixth Element Capital in January 2023. However, the work described in this paper was undertaken during the time frame when the license for SRA737 was initially held by Sixth Element Capital and then Sierra Oncology.

Studies have shown SRA737 to be an effective CHK1 inhibitor in the preclinical setting, abrogating etoposide-mediated G2/M arrest in HT-29 cells, with preclinical activity also being demonstrated in RAS NSCLC and Eμ-MYC driven B-cell lymphoma models [33]. Recently SRA737 has undergone a phase 1/2 clinical trial both alone (NCT02797964) and in combination (gemcitabine plus cisplatin or gemcitabine alone; NCT02797977).

Given the high frequency of genetic anomalies in the CRC and NSCLC setting that render these cancer cells potentially susceptible to CHK1 inhibition, the purpose of the present study was to investigate the *in vitro* effects of the novel CHK1 inhibitor SRA737 as a single agent on pairs of NSCLC and CRC cell lines of different genetic backgrounds (notably of different *TP53* status); specifically, on the level of DNA damage induced and the subsequent effect on cell proliferation/viability. Alkali comet and phosphorylated H2A.X variant histone (γH2AX)-foci assays were used to assess mostly DNA SSB and double strand break (DSB) damage, respectively, and cell-counting/trypan blue staining was used to assess cell proliferation/viability.

## Materials and methods

### Cells, reagents & culture conditions

All cell lines [the NSCLC cell lines H23 (*TP53* mutant [MUT]) and A549 (*TP53* wild-type [WT]) and the CRC cell lines HT29 (*TP53* MUT) and HCT116 (*TP53* WT)] were purchased from the American Type Culture Collection. A549 cells were cultured in Dulbecco’s modified eagle medium (DMEM)-high glucose media, whilst H23 cells were cultured in Roswell Park Memorial Institute (RPMI) 1640 medium. HCT116 cells were cultured in McCoy’s media and HT29 cells were cultured in DMEM media +1% Glutamax (1×). All media were supplemented with 10% (v/v) fetal bovine serum (FBS) and all cells were cultured at 37°C in a humidified atmosphere [95% air + 5% CO_2_] and maintained at a low passage (< 50). SRA737 was obtained via the Cancer Research UK Centre for Drug Development from The Institute of Cancer Research Cancer Therapeutics Unit (UK).

### Alkaline comet assay

For the alkaline comet assay (ACA) assessment of SRA737-induced DNA damage, cells were treated in their own media with 0 [dimethylsulfoxide (DMSO)-only, final concentration 0.1% v/v], 0.1 µmol/L, 0.5 µmol/L, 1 µmol/L or 5 µmol/L SRA737 for 24 h, under 5% CO_2_ at 37°C.

Following treatment, the cells were harvested and *ca.* 30,000 SRA737-treated or untreated (control) cells were separately mixed with 200 µL of molten (cooled to 37°C) low melting point agarose [0.6% in phosphate buffered saline (PBS)]. Immediately after mixing, 2× 80 µL aliquots were taken and separately placed on the clear glass section of a double frosted end microscope slide pre-coated with dried normal melting point agarose [whereby the microscope slides were dipped in molten normal melting point agarose (1% prepared in double distilled water, ddH_2_O) and left to dry overnight at room temperature]. Coverslips (22 mm × 22 mm) were immediately placed onto the two agarose droplets (enabling even distribution of the gels) and the slides were then placed onto a metal tray on ice for 20 min to allow the gels to set, then the coverslips removed.

The levels of SRA737-induced DNA damage were assessed using the alkaline version of the comet assay with slight modifications of the original method [34]. Briefly, slides bearing the treated and untreated cells (embedded in 22 mm × 22 mm 0.6% low melting point agarose; prepared as described above), were incubated overnight at 4°C in lysis buffer [2.5 mol/L NaCl, 100 mmol/L ethylene diamine tetraacetic acid disodium salt (Na_2_EDTA), 10 mmol/L Trisbase, 1% Triton X-100, pH 10]. Following lysis, the slides were washed twice for 10 min with ice-cold double distilled water (ddH_2_O) and then transferred to the electrophoresis tank. Ice-cold electrophoresis buffer (300 mmol/L NaOH and 1 mmol/L Na_2_EDTA in ddH_2_O, pH 13) was added to the tank and the slides incubated for 20 min and then subjected to electrophoresis [20 min at 30 V (*ca.* 0.8 V/cm), 300 mA] in the same buffer. Slides were then incubated in neutralisation buffer (0.4 mol/L Tris Base in ddH_2_O, pH 7.5) for 20 min, washed twice for 10 min with ddH_2_O and allowed to dry at 37°C. Once dry, slides were rehydrated with ddH_2_O for 20 min then stained with approximately 1 mL propidium iodide (PI) diluted in ddH_2_O (2.5 µg/mL) for 25 min, washed twice for 10 min with ice-cold ddH_2_O and then dried at 37°C; after drying, the slides were stored in a light-tight box prior to analysis. For comet visualisation, a drop of ddH_2_O was placed onto the centre of each slide gel and the gel then covered with a fresh cover slip.

Comets were visualised at ×200 magnification using an Olympus BH-2-RFL-T2 fluorescent microscope fitted with an excitation filter of 515–535 nm and a 590 nm barrier filter, and images were captured using a high performance CCDC camera (COHU MOD 4912-5000/0000) connected to a computer running COMET IV software (Perceptive Instruments/Instem). Comets were captured and scored by randomly selecting 50 comets from the centre of each gel. COMET IV software calculated percent tail DNA (%), tail moment [arbitrary units (a.u.)], Olive tail moment (a.u.) and tail length (µm) automatically and produces a spreadsheet of data for each slide with the results presented as the combined mean ± standard error of mean (SEM) of the 100 analysed comets.

For the H_2_O_2_ treatment of cells, to generate “damage-positive” controls, *ca.* 100,000 cells were treated with 50 µmol/L H_2_O_2_ (Sigma-Aldrich) in serum-free RPMI-1640 media in 6-well plates, protected from light for 30 min on ice. Preliminary studies conducted in this lab demonstrated an inhibitory effect on H_2_O_2_-induced DNA damage in cells when using cell media containing pyruvic acid (i.e., DMEM; data not shown); this is supported by the finding of Long and Halliwell [35] which reports that media containing pyruvate serves to scavenge hydrogen peroxide. So, for the treatment of the NSCLC and CRC cells with H_2_O_2_, cell-specific media was removed and the cells then washed twice with pre-warmed PBS (pH 7.4) and treated with serum-free RPMI-1640 media containing H_2_O_2_ (50 µmol/L) on ice for 30 min, protected from light. After treatment, the cells were harvested and prepared for comet assay analysis as described above.

### γH2AX-Foci assay

For the γH2AX foci assessment of SRA737-induced DNA damage, cells were seeded and grown on sterile cover slips placed on the bottom of wells of six-well plates containing *ca.* 50,000 cells per well and 2 mL of their own media and incubated overnight under 5% CO_2_ at 37°C. The following day the overnight media was removed and the coverslip-attached cells were treated with their own media containing 0 (DMSO-only, final concentration 0.1% v/v), 0.1 µmol/L, 0.5 µmol/L, 1 µmol/L or 5 µmol/L SRA737 for 24 h under 5% CO_2_ at 37°C.

Following treatment, the coverslip-attached cells in the six well plates were washed twice with 2 mL of ice-cold PBS and fixed with 1 mL 100% methanol and kept at –20°C overnight. The cells were then washed twice with 1 mL of PBS for 20 min (10 min per wash) and then incubated for 15 min in KCM blocking buffer [0.12 mol/L KCl, 20 mmol/L NaCl, 10 mmol/L Tris-HCl and 1mmol/L ethylene diamine tetraacetic acid (EDTA) with 2% (w/v) of bovine serum albumin (BSA), 10% (w/v) normal goat serum, 10% (w/v) goat milk powder with 0.1% (v/v) Triton X-100 added fresh]. Following the removal of the KCM blocking buffer, the cells were then treated with 150 μL of primary anti-phosphohistone H2A.X variant histone (H2AX; Ser139) antibody (Clone JBW301, Mouse Monoclonal Antibody; Thermo Fisher Scientific) diluted in 1× KCM blocking buffer at a ratio 1:200 and incubated at room temperature for 2 h on a shaker. Within each well, the coverslips were next gently washed four times with 1 mL of KCM washing buffer [0.12 mol/L KCl, 20 mmol/L NaCl, 10 mmol/L Tris-HCl and 1 mmol/L EDTA with 0.1% (v/v) Triton X-100 added fresh]. The coverslips were then treated with 150 μL of secondary antibody [A21121 Alexa Fluor 488 Goat Anti-mouse immunoglobulin G (IgG); Thermo Fisher Scientific], diluted in blocking buffer at a ratio of 1:200. The coverslips were placed in the dark at room temperature for 1 h to prevent further damage to the DNA. The coverslips were then washed 4 times with washing buffer (5 min per wash). Following the final wash, a drop of SlowFade^®^ Gold antifade reagent (Thermo Fisher Scientific) with 4’,6-diamidino-2-phenylindole (DAPI; 10 μL) was dispensed onto the clear glass section of a double frosted end microscope slide and then carefully, each coverslip was removed from its well and placed (sample facing down) onto the drop of SlowFade^®^ Gold antifade reagent; the cells being between the cover slip and the slide surface. Finally, the coverslip was secured by using nail polish on all sides, and the slides were then kept at 4°C prior to image viewing and analysis.

The foci were visualised at ×40 magnification power and captured using an Olympus Cytological imaging system together with Data Base Program software. An automated meta-system was used to assess 6 slides each time. Images of clear γH2AX foci were captured using a 485 μmol/L filter, whereas the number of DAPI stained nuclei images were captured using a DAPI filter. Up to 100 randomly chosen cells per slide were scored and analysed by Image J software [WCIF Image J version 1.42, available from the research services branch National Institutes of Health (NIH)]. As a part of the software’s functioning, γH2AX foci and nuclei numbers were counted automatically. After the exclusion of cells with more than one nucleus, the average number of γH2AX foci per cell (DAPI nuclei) was obtained by dividing the total number of γH2AX foci by the total number of cells per field.

For the radiation treatment of the cells, to generate “damage-positive” controls, an XSTRAHL RS320 Irradiation cabinet was used to expose the coverslip-attached cells in their own media in six well plates to 2 gray (Gy) of 320 kV X-rays on ice; the plates were then incubated for 30 min under 5% CO_2_ at 37°C. After treatment, the cells were assayed for γH2AX foci as described above.

### Cell proliferation & viability assay

For the assessment of SRA737-induced effects on cancer cell proliferation and viability, cells were treated in their own media with either DMSO-only (0.1% v/v) or 1 µmol/L or 5 µmol/L SRA737 for 2–4 days under 5% CO_2_ at 37°C. Following harvesting, equal volumes (20 µL) of cell suspension and trypan blue stain were thoroughly mixed and then approximately 10 µL of the mixture was pipetted into a haemocytometer chamber (Neubauer chamber) under a cover slip. Cells were then counted as either viable cells (white) or non-viable cells (blue) in four 1 mm^2^ areas of the chamber; this was performed within 2 min to avoid recording false negative results. The number of viable cells per 1 mL of cell suspension was obtained by multiplying the average number of white cells per 1 mm^2^ area by the dilution factor and by 10^4^ (the conversion of 0.1 mm^3^ to 1 mL). The viable percentage was calculated by dividing the number of viable cells by the total number of cells (viable plus non-viable cells) and multiplied by 100.

### Statistical analysis

Statistical differences between the indicated replicate measures were assessed by a two-tailed unpaired *t*-test with Welch’s correction, with the extent of the significant difference indicated by the following categorisations: ^*^ *P* < 0.05, ^**^ *P* < 0.01, ^***^ *P* < 0.001, and ^****^ *P* < 0.0001.

## Results

**Figure 1:**
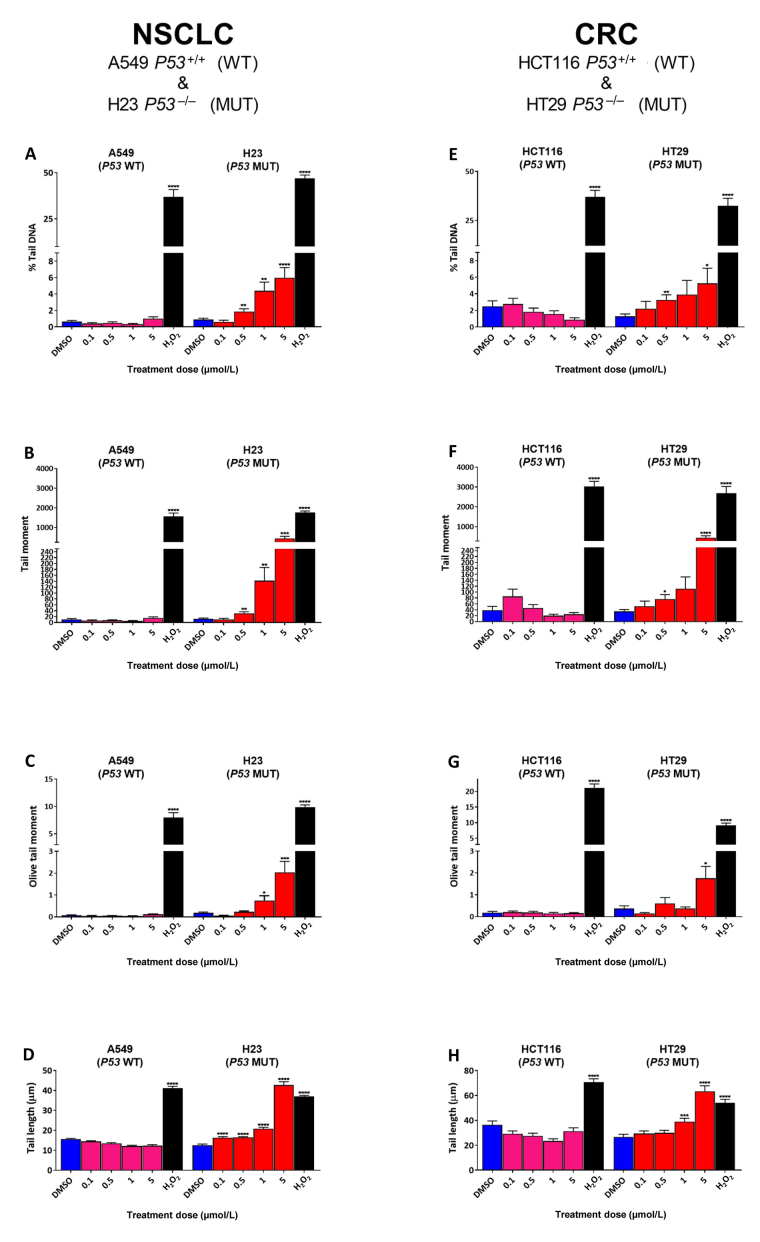
Comet assay analysis of DNA damage induced by SRA737 in A549 & H23 NSCLC cells and HCT116 & HT29 CRC cells. NSCLC A549 (*TP53* WT) & H23 (*TP53* MUT) cells (A–D) and CRC HCT116 (*TP53* WT) & HT29 (*TP53* MUT) cells (E–H) were treated in their own media with 0 (vehicle-only; DMSO 0.1% v/v), 0.1 µmol/L, 0.5 µmol/L, 1 µmol/L or 5 µmol/L SRA737 for 24 h under 5% CO_2_ at 37°C. Following treatment, the cells were harvested/prepared and analysed by the ACA as outlined in the “Materials and methods” section. The extent of damage formation was determined as the drug-induced increase in the scored comet’s % tail DNA (%; A & E), tail moment (a.u.; B & F), Olive tail moment DNA (a.u.; C & G) & tail length (µm; D & H). A hundred comets were scored for each treatment condition and the results presented as the mean ± SEM for the respective scores. DMSO-only treated cells represent untreated/negative controls whilst 50 µmol/L H_2_O_2_-treated cells represent damage-positive controls. Statistical differences between the SRA737- or H_2_O_2_-treated cells and the corresponding DMSO-controls were assessed by a two-tailed unpaired *t*-test with Welch’s correction: ^*^ *P* < 0.05, ** *P* < 0.01, ^***^ *P* < 0.001, and ^****^ *P* < 0.0001

To determine the effect of SRA737 treatment on “total” DNA strand break damage formation, both NSCLC H23 (*TP53* MUT) and A549 (*TP53* WT) cells and CRC HT29 (*TP53* MUT) and HCT116 (*TP53* WT) cells were incubated for 24 h with 0.1 µmol/L, 0.5 µmol/L, 1 µmol/L or 5 µmol/L of SRA737 and then analysed using the ACA. The results (Figure 1) are presented as the mean ± SEM of the 100 analysed comets. The alkaline version of the comet assay detects strand break damage resulting from both induced SSBs and DSBs, plus strand breaks resulting from the formation of alkali-labile sites (including abasic sites and certain base lesions) [34, 36, 37]. DMSO-treated cells represent non-drug/vehicle-only treated controls, whilst H_2_O_2_-treated cells represent damage-positive controls. The results for the NSCLC cell lines are shown in Figure 1A–D, whilst the results for the CRC cell lines are shown in Figure 1E–H with the extent of comet formation (reflecting DNA damage induction) being reported using the four most commonly used comet parameters; % tail DNA (Figure 1A and Figure 1E), tail moment (Figure 1B and Figure 1F), Olive tail moment (Figure 1C and Figure 1G) and tail length (Figure 1D and Figure 1H).

All four comet parameters revealed clear concentration-dependent increases in comet formation for both of the H23 and H29 *TP53* MUT cells, with the increase being consistently significant for the highest concentration of SRA737 used (5 µmol/L) in both cell lines for all the four comet parameters. In contrast, there was no significant increase in comet formation noted for either of the *TP53* WT cells (A549 and HCT116), indicating no ACA-detectable DNA damage being induced in these cells upon treatment with SRA737.

**Figure 2:**
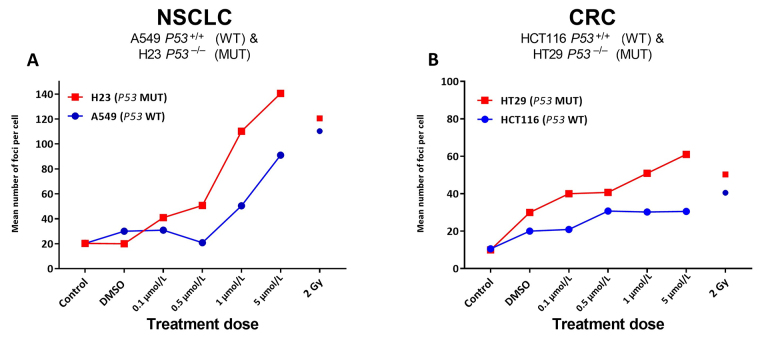
γH2AX foci analysis for the detection of DNA double-strand break damage in A549 & H23 NSCLC cells and HCT116 & HT29 CRC cells following treatment with SRA737. NSCLC A549 (*TP53* WT) & H23 (*TP53* MUT) cells (A) and CRC HCT116 (*TP53* WT) & HT29 (*TP53* MUT) cells (B) were seeded overnight on coverslips and treated in their own media with 0 (vehicle-only; DMSO 0.1% v/v), 0.1 µmol/L, 0.5 µmol/L, 1 µmol/L or 5 µmol/L SRA737 for 24 h under 5% CO_2_ at 37°C. Following treatment, the cells were prepared and γH2AX foci counted as outlined in the “Materials and methods” section. The results are presented as the mean foci number per cell recorded for up to 100 cell nucleic from 10–15 fields of view per slide. Untreated and DMSO-only treated cells represent untreated/negative controls whilst radiation treated cells (2 Gy) represent damage-positive controls

Given the significant increases noted for ACA-detected SRA737-induced damage in the *TP53* MUT cells (Figure 1), next, γH2AX foci levels were assessed as a marker of possible SRA737-induced DSB damage in the pairs of NSCLC and CRC cell lines (Figure 2). Cells were seeded overnight on coverslips and treated for 24 h with 0.1 µmol/L, 0.5 µmol/L, 1 µmol/L or 5 µmol/L SRA737. Following treatment, the cells were prepared and γH2AX foci detected and counted from 10–15 recorded fields of view, and the results are presented as the mean foci number for at least 100 cell nuclei per condition studied. Untreated and DMSO-treated cells represent untreated and non-drug/vehicle-only treated controls, respectively, whilst 2 Gy irradiated cells represent damage-positive controls. The results for the NSCLC A549 & H23 cells are shown in Figure 2A whilst the results for the CRC HCT116 & HT29 cells are shown in Figure 2B.

SRA737 treatment induces higher levels of γH2AX foci in a dose dependant fashion, in the *TP53* MUT H23 NSCLC cells and HT29 CRC cells compared to the corresponding *TP53* WT A549 NSCLC and HCT116 CRC cells. As expected [38], radiation induces near-equivalent DSB levels in the respective *TP53* WT and MUT pairs, though this was noted to be higher for the NSCLC cells and generally slightly higher in the respective *TP53* MUT cells.

**Figure 3:**
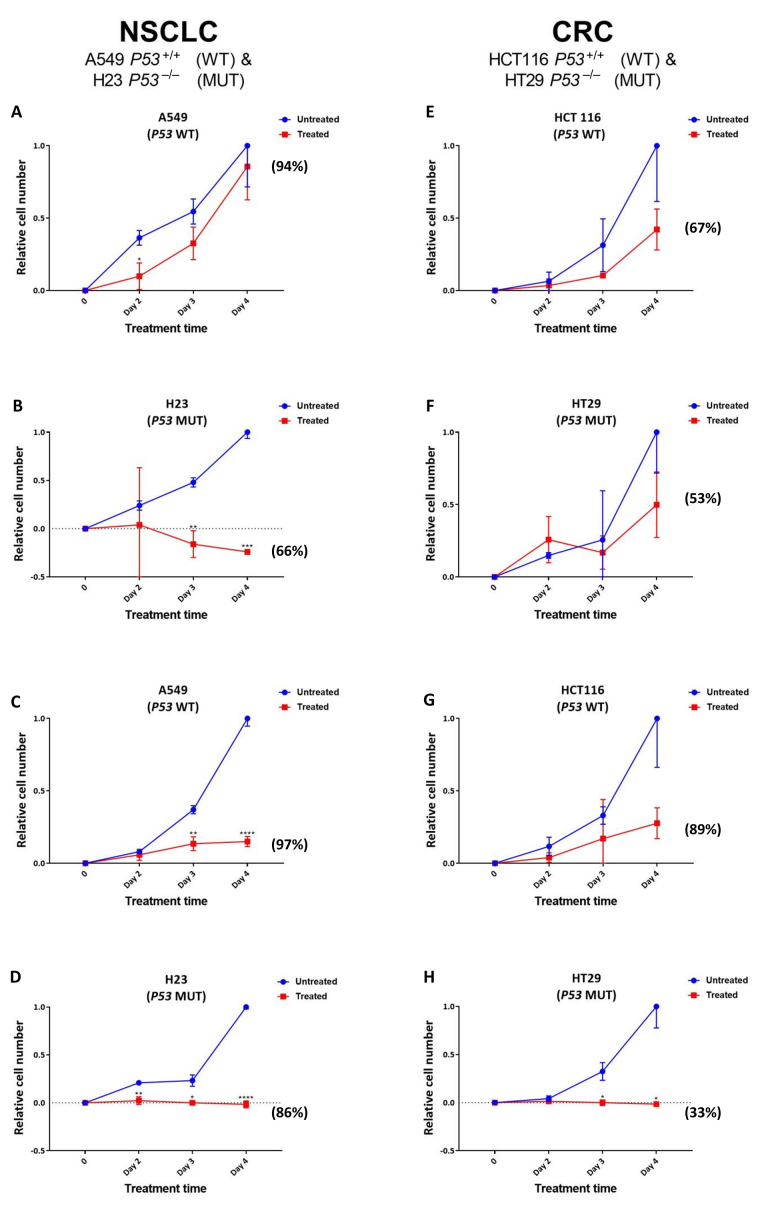
Proliferation/viability analysis of A549 & H23 NSCLC cells and HCT116 & HT29 CRC cells following treatment with SRA737. NSCLC A549 (*TP53* WT) & H23 (*TP53* MUT) cells (A–D) and CRC HCT116 (*TP53* WT) & HT29 (*TP53* MUT) cells (E–H) were treated in their own media with 0 (vehicle-only; DMSO 0.1% v/v), 1 µmol/L (A, B & E, F) or 5 µmol/L (C, D & G, H) SRA737 for 2–4 days under 5% CO_2_ at 37°C. Following treatment, the cells were harvested, stained (Typan Blue) and counted as outlined in the “Materials and methods” section. The results are presented as the mean ± SD of the relative increase in cell number (relative to the increase noted on day 4 for the untreated cells) from three determinations. Statistical differences between the SRA737-treated cells and the corresponding DMSO-controls were assessed by a two-tailed unpaired *t*-test with Welch’s correction: ^*^ *P* < 0.05, ^**^ *P* < 0.01, ^***^ *P* < 0.001 & ^****^ *P* < 0.0001. The values in parenthesis represent the % viability of the cells after four days of treatment; the corresponding viability levels noted for the non-drug/vehicle-only treated cells were all between 95–100%

Given the greater increase noted for SRA737-induced γH2AX foci levels in the *TP53* MUT cells (Figure 2), reflecting potentially lethal DSB damage formation [39], next, the effect of SRA737 on NSCLC and CRC cell proliferation/viability was assessed (Figure 3). NSCLC H23 and A549 cells and CRC HT29 and HCT116 cells were incubated with either 1 µmol/L or 5 µmol/L SRA737 for 2–4 days, then their relative cell number and viability was assessed via cell counting together with trypan blue exclusion. The latter assay is based on the circumstance that intact/functional cell membranes of live cells prevent Trypan blue from entering into the cell, so live/viable cells remain unstained (white/clear); however, the stain traverses the cell membrane of dead cells, which become a distinctive blue colour. DMSO-treated cells represent non-drug/vehicle-only treated controls and their increased cell number is taken to reflect normal proliferation. The results for the NSCLC cell lines are shown in Figure 3A–D, whilst the results for the CRC cell lines are shown in Figure 3E–H, with the observed cell numbers for both the untreated and SRA737-treated cells being reported relative to the number of cells noted for the untreated cells at day 4. The results following 1 µmol/L treatments are shown in Figure 3A, Figure 3B, Figure 3E, and Figure 3F; the results following 5 µmol/L treatments are shown in Figure 3C, Figure 3D, Figure 3G, and Figure 3H.

Clearly, there was an agent-dependent decrease in cell proliferation following the treatment of both the *TP53* MUT and WT NSCLC and CRC cells, with the decrease in cell growth following 5 µmol/L treatment compared the growth of the untreated cells being significant at days 3–4 for the A549, days 2–4 for H23 and days 3–4 for H29. There was clearly a greater relative loss of cell proliferation noted following 5 µmol/L treatment for the *TP53* MUT cells compared to the *TP53* WT cells, with the *TP53* MUT cells H23 (Figure 3D) and HT29 (Figure 3H) showing no increase in cell number over the 4 days of treatment. Furthermore, following the 1 µmol/L treatment of the NSCLC cell pair (Figure 3A and Figure 3B), there is clearly a greater loss of proliferation noted for the *TP53* MUT cell H23 (Figure 3B) upon treatment, compared to the *TP53* WT cell A549 (Figure 3A); the decrease in cell growth being highly significant at days 3–4 for the H23 cells, compared the growth of the untreated cells. In contrast, there was no significant difference noted for the effect of 1 µmol/L SRA737 on the CRC cell lines HCT116 and H29, with the SRA737-treated cells showing similar non-significant decreases in cell proliferation compared to the non-treated cells (Figure 3E and Figure 3F).

The above scenario also extends to the assessment of cell viability as measured by Trypan blue exclusion. Following both 1 µmol/L and 5 µmol/L treatments, a greater loss of cell viability was noted for the *TP53* MUT H23 and HT29 cells after 4 days of treatment, than for the *TP53* WT A549 and HCT116 cells (see the % viability values in parenthesis in Figure 3); though this was not noted to be strictly dose dependant. The viability levels noted for the non-drug/vehicle-only treated cells were all between 95–100%.

## Discussion

Oncogene activation or mutations in either repair and cell-cycle machinery, plus other genomic alterations, can all lead to replication stress in cancer cells. This can result in elevated damage and instability, leading to an increased CHK1 dependency for survival, particularly for cells with dysfunctional P53. Therefore, CHK1 inhibition may be synthetically lethal in cancer cells with high replicative stress. SRA737 is a new inhibitor of CHK1 that has recently undergone phase 1/2 testing both as a single agent and in combination [40, 41].

Two common tumour types with a high occurrence of genetic aberrations considered causative of replication stress, and therefore increased CHK1 dependency, include metastatic colorectal and NSCLC. For NSCLC, mutations of the *TP53* gene occur in about 50% of cases [42, 43], with the majority of studies indicating that alterations in *TP53* are associated with a poor prognosis [44]. Furthermore, *TP53* mutation occurs in approximately 40–50% of sporadic CRC [45], with the mutation status being closely related to progression and outcome; patients with MUT *TP53* appear more chemo-resistance and have poorer prognosis than those with WT *TP53* [46]. Consequently, the present study was undertaken to assess the *in vitro* effect of SRA737 on pairs of NSCLC (A549 & H23) and CRC (HCT116 & HT29) cancer cell lines, with each pair processing a cell line with MUT *TP53* [H23: bearing a mutation at codon 246 (C → G; Ile → Met); HT29: bearing a mutation at codon 273 (G → A; Arg → His)]. Three of the cell lines (A549, H23 & HCT116) have activating mutations of the growth promoting oncogene *KRAS*, whilst HT29 cells, although *KRAS* WT, have an activating *BRAF* mutation (*V600E*), which triggers the mitogen-activated protein kinase (MAPK) pathway [47]; consequently, all four cell lines are considered prone to replicative stress. Investigation of the *in vitro* effects of SRA737 included an assessment of DNA strand break damage formation (including potentially lethal DSB damage formation), as assessed by ACA analysis and γH2AX-foci counting (Figure 1 and Figure 2, respectively), plus the subsequent effect on cell proliferation/viability (Figure 3).

Clearly, SRA737 treatment of the *TP53* MUT H23 NSCLC and HT29 CRC cells led to significant increases in ACA-detectable strand break DNA damage compared to the *TP53* WT A549 NSCLC & HCT116 CRC cells (Figure 1); this being consistently and significantly higher for the highest concentration of SRA737 used, for both *TP53* MUT cells. In contract, no significant increase was noted for either of the *TP53* WT cells, even for the highest concentration of SRS737 used. All four commonly recorded comet parameters are reported on account of the relatively low comet response being noted for the SRA737-treated cells; note the far greater comet responses being reported by % tail DNA (Figure 1A and Figure 1E), tail moment (Figure 1B and Figure 1F) and Olive tail moment (Figure 1C and Figure 1G) for the H_2_O_2_-treated cells (representing “damage-positive” controls) versus the SRA737-treated cells; the exception being tail length (Figure 1D and Figure 1H) which is regarded as a sensitive comet measure of damage, but which saturates at low damage levels [48].

Matching the responses noted for the ACA analysis, SRA737 treatment led to clear increases in the number of γH2AX-foci (reflecting likely DSB formation) for the *TP53* MUT NSCLC and CRC cells, with consistently lower foci numbers being noted for the corresponding *TP53* WT cells (Figure 2). The observation of increases in ACA-detectable damage together with increases in γH2AX-foci numbers is in line with the expectation of the SRA737-mediated inhibition of CHK1 in a P53 deficient setting; whereby, increased oncogene-induced firing of replication encountering elevated endogenous damage results in the accumulation of stalled replication forks [1, 49] which, via subsequent fork collapse, leads to increased DSB damage formation.

The observation of higher SRA737-induced γH2AX-foci numbers following treatment of the *TP53* MUT NSCLC and CRC cells was indicative of SRA737 having a potentially greater anti-proliferative effect in the MUT cells than in WT cells, via the formation of lethal DSB damage [39]. This was supported via the assessment of SRA737 treatment on cell proliferation and cell viability (Figure 3). SRA737 had a greater anti-proliferative effect on the *TP53* MUT H23 and HT29 cells than on the WT cells. This also included evidence of elevated cell killing in the *TP53* MUT cells as indicated by the “lower-than-seeded” cell numbers being noted for H23 following both 1 µmol/L and 5 µmol/L treatments and for HT29 cells following 5 µmol/L treatment, together with a greater loss of cell viability being noted for the *TP53* MUT H23 and HT29 cells than for the *TP53* WT A549 and HCT116 cells following treatment. Again, this is consistent with the expected outcome of SRA737’s inhibition of CHK1 in a P53 deficient setting; whereby, increased replication fork stalling and collapse leads to increased levels of lethal DSB damage.

Whilst there was no clear difference in the level of ACA assessed induced damage between the NSCLC and CRC cell pairs (Figure 1), there was a higher level of potentially-lethal DSB damage, as assessed by higher γH2AX-foci counts, induced in the NSCLC cell pair (*ca.* 120–70 foci per cell nuclei for the highest dose of SRA737 used) compared to the CRC cell pair (*ca.* 50–20 foci per cell nuclei; Figure 2). This in turn may account for the greater sensitivity of the *TP53* MUT NSCLC H23 cell line towards lower dose SRA737 than the *TP53* MUT CRC HT29 cell line (Figure 3B *vs.* Figure 3F).

The current study indicates that CHK1 inhibition alone may deliver therapeutic activity in a P53 deficient background for NSCLC and CRC; both common tumour types of high unmet medical need, particularly in the advanced setting. The data reported (notably the greater levels of agent-induced DNA damage and anti-proliferative activity in the *TP53* MUT cells) matches the expected mechanism of action of SRA737 in such a genetic background. For the *TP53* MUT H23 and HT29 cells, oncogene-induced increases in the firing of replication origins and a consequential depletion of replication protein A (RPA) and dNTPs results in the accumulation of stalled replication forks [1, 49]; and with their G1/S checkpoint compromised, this, in turn, leads to a greater reliance CHK1, and the associated the G2/M and S checkpoints, to stall mitotic progression and allow for DNA repair and so to prevent fork collapse and DNA damage. Accordingly, with the G1/S checkpoint compromised, the SRA737 inhibition of CHK1 delivers a state of synthetic lethality in P53 deficient cells, leading to irreparable levels of DNA damage, mitotic catastrophe and subsequent tumour cell death. In contrast, the *TP53* WT A549 and HCT116 cells, with their intact ATM, CHK2 and P53 signalling, are still able to enter cell-cycle arrest and repair damage using the functioning G1/S checkpoint, thereby being more rescued from cytotoxicity. However, a limitation of the present study is that, instead of analysing isogenic cells, the study compared different established cell lines, which differ from each other not only by *TP53* status but also probably many other features. However, the results generated are very much in accord with expectation, with the analysed inhibitor (SRA737) demonstrating higher activity towards *TP53* mutated compared to *TP53* WT cells.

The findings of the current study are supported by other studies that have shown CHK1 inhibitors to be effective as single agents in defined tumour genetic backgrounds [50–52]; such as those with activating *KRAS* or *MYC* mutations, loss-of-function *ATM* or *TP53* mutations, or with repair gene defects. CHK1 inhibition has been demonstrated in MYC driven tumours and in cancers of heightened replicative stress [51–54]. Furthermore, SRA737 as a single agent has been studied in various of murine xenograft models bearing genetic alterations that render tumour cells sensitive to CHK1 inhibition, including a spontaneous model of *N-MYC*-driven neuroblastoma [51] plus an MOLM13 xenograft model of AML and an orthotopic solid tumour model of triple negative breast cancer (unpublished supporting data: Clinical Study Protocol No. SRA737-01, Sierra Oncology, Inc. available at: https://clinicaltrials.gov/ct2/show/NCT02797964); DDR marker analysis confirming this activity to be due to SRA737’s anticipated mechanism of action.

Whilst the exact molecular signature for sensitivity to CHK1 inhibitor monotherapy is still under investigation, the findings of this study suggest that P53 status/functioning is a likely key factor in determining SRA737 efficacy in the NSCLC and CRC setting. Furthermore, these findings support the concept that tumours with heightened genomic instability coupled with replicative stress will display enhanced sensitivity towards CHK1 inhibitors, both as single agents and in combination with DNA damaging agents or other DDR inhibitors.

Recently Jones et al. [40] reported data from a phase 1/2 trial of SRA737 in combination with a novel, lower dose of gemcitabine (LDG) compared to standard of care (ClinicalTrials.gov identifier NCT02797977, EudraCT Number: 2015–004467–36). Treatment of patients with SRA737 in combination with LDG was found to be well tolerated (the toxicities noted seldom led to the cessation of treatment) and resulted in lower myelotoxicity compared to standard treatment or in combination with other CHK1 inhibitors. Tumour responses were noted in anogenital and other solid tumours, with an objective response rate in anogenital cancer of 25%. Partial tumour responses were also observed for a variety of other cancers, indicating SRA737’s anti-cancer activity in multiple indications. Many of the observed responses were associated with alterations in the Fanconi anaemia (FA)/BRCA network and factors involved in the repair of replication forks, and in tumours of intermediate to high mutational burden; providing clinical evidence of the effectiveness of LDG plus SRA737, justifying further study.

However, despite the positive reports of CHK1 inhibitors preclinically, overall, trial outcomes of CHK1 inhibitors have to date not lived up to expectation. This has included failure to substantially improve the standard treatment [40, 55], off-target effects [56], toxicity [57] and other contrary effects [58] or trial termination due to business reasons [59]. In addition, evidence of independence towards P53 has been observed in some studies and trials [59]. Thus, for CHK1 inhibition to ultimately progress, further new factors/biomarkers affecting the efficacy of CHK1 inhibitors need to be identified and translated to the clinic; with the perhaps most useful being those that allow for inhibitor-specific patient stratification criteria.

## Abbreviations

a.u.: arbitrary units
ACA: alkaline comet assay
ATM: ataxia–telangiectasia mutated
ATR: ataxia-telangiectasia and Rad3-related
BRAF: v-raf murine sarcoma viral oncogene homolog B1
BRCA1: breast cancer gene 1
CHK1: checkpoint kinase 1
CHK2: checkpoint kinase 2
CRC: colorectal cancer
DAPI: 4’,6-diamidino-2-phenylindole
ddH_2_O: double distilled water
DDR: DNA damage response
DMEM: dulbecco’s modified eagle medium
DMSO: dimethylsulfoxide
DSB: double strand break
EDTA: ethylene diamine tetraacetic acid
G1: G1 phase
G2: G2 phase
Gy: gray
H_2_O_2_: hydrogen peroxide
KRAS: Kirsten rat sarcoma
LDG: lower dose gemcitabine
Na_2_EDTA: ethylene diamine tetraacetic acid disodium salt
M: M phase
MUT: mutant
MYC: myelocytomatosis
NSCLC: non-small cell lung cancer
P53: tumour suppressor protein 53
PBS: phosphate buffered saline
PI: propidium iodide
RAS: rat sarcoma
RPMI: Roswell Park Memorial Institute
S: S phase
SEM: standard error of mean
SSB: single strand break
TP53: tumour protein 53
WT: wild-type
γH2AX: phosphorylated H2A.X variant histone

## Declarations

### Availability of data and materials

The raw data supporting the conclusions of this manuscript will be made available by the authors, without undue reservation, to any qualified researcher.

### Copyright

© The Author(s) 2023.
